# LongQC: A Quality Control Tool for Third Generation Sequencing Long Read Data

**DOI:** 10.1534/g3.119.400864

**Published:** 2020-02-10

**Authors:** Yoshinori Fukasawa, Luca Ermini, Hai Wang, Karen Carty, Ming-Sin Cheung

**Affiliations:** King Abdullah University of Science and Technology (KAUST), Core Labs, Thuwal, Makkah, Saudi Arabia, 23955-6900

**Keywords:** Quality control, Long read, third generation sequencers, PacBio, Oxford Nanopore

## Abstract

We propose LongQC as an easy and automated quality control tool for genomic datasets generated by third generation sequencing (TGS) technologies such as Oxford Nanopore technologies (ONT) and SMRT sequencing from Pacific Bioscience (PacBio). Key statistics were optimized for long read data, and LongQC covers all major TGS platforms. LongQC processes and visualizes those statistics automatically and quickly.

Short read sequencing technologies have changed the paradigm in biology for the past decade. Recently, TGS have emerged and provided remarkably long but relatively error-prone reads from single molecules. There are standard quality control (QC) methods for short reads such as FastQC (https://www.bioinformatics.babraham.ac.uk/projects/fastqc/) or PRINSEQ (Schmieder and Edwards 2011); however, these methods have not been fully optimized for characterizing long error-prone read. Because the scientific community is fast adopting TGS, it is worth reconsidering the applicability and suitability of such methods for long read data. Unique properties of long read data require different QC statistics to describe the different characteristics.

Phred quality score is a widely used measurement to assess the quality of sequencing data. Unfortunately, this is not applicable for the PacBio Sequel system which no longer provides meaningful Phred scores. Without Phred scores as an indicator of basecalling confidence, coverage becomes a crucial criterion to achieve reliable consensus sequences from the dataset. This also affects the recently released NanoPack (De Coster *et al.* 2018), mainly developed for ONT dataset. Despite capability of PacBio uBam loading, NanoPack cannot fully assess data due to the lack of Phred score. Other QC tools available within the ONT community are only suitable for ONT because they summarize platform specific statistics using meta-data generated by ONT systems (Loman and Quinlan 2014; Watson *et al.* 2015; Lanfear *et al.* 2019). Such tools can be applied before a full analysis; however, they are susceptible to format change of meta-data. In fact, changes in raw data format (Fast5) and basecalling programs have been frequent for ONT. Earlier ONT QC tools are no longer applicable with the latest Fast5 format. Additionally, new ONT basecallers such as Scrappie or Bonito skip Phred score calculation like Sequel; therefore, development of a QC tool that is insensitive to file formats or Phred score availability is beneficial.

An alternative QC approach is to apply read polishing tools. DASCRUBBER, a suite developed for scrubbing PacBio reads (https://github.com/thegenemyers/DASCRUBBER), although not designed specifically for QC *per se*, can be used for quality assessment. Under DASCRUBBER framework, all-*vs.*-all comparison of reads is executed to find and trim erroneous segments. The whole process can take hours on a workstation for completeness and precision. Additionally, limited number of supported file formats, running multiple commands in the suite with different parameter options, and lack of explicit means to judge data quality could pose a challenge for inexperienced users.

It has been reported that ONT reads from low quality pores can be random, even though they sometimes show misleadingly high quality scores (Mojarro *et al.* 2018). These reads are artificially produced and have no biological relevance to the sample. In fact, each TGS dataset can contain a certain amount of “non-sense reads”, which we define as unique reads that cannot be mapped onto sequences of any other molecules in the same library. We thus propose that the fraction of non-sense reads contained in a dataset can be used as a robust indicator of sequencing quality.

Here we report a new QC pipeline for TGS data named LongQC (Long read Quality Control). LongQC is a computationally efficient, platform-independent QC tool to spot issues before a full analysis. The tool visualizes statistics designed for erroneous long read data to highlight potential problems originated from the biological samples as well as those introduced at the sequencing stage. It supports major TGS file formats. LongQC relies on k-mer based internal overlaps and skips alignment; therefore, it operates efficiently without reference genomes.

## Materials and Methods

### Datasets

*Escherichia coli* strain K-12 genome was sequenced on PacBio RS-II, PacBio Sequel and ONT MinION. Public datasets were retrieved from published studies (Michael *et al.* 2018; Mojarro *et al.* 2018) and PacBio DevNet (https://github.com/PacificBiosciences/DevNet/wiki/Datasets). Simulated datasets were generated using PBSIM version 1.0.4 and NanoSim version 2 (Ono *et al.* 2013; Yang *et al.* 2017). See supplementary information for further details.

### Quantification and estimation of actual fraction of non-sense reads

We mapped actual reads from PacBio RS-II, PacBio Sequel, and ONT MinION onto known reference genomes using minimap2 ver. 2.6 (Li 2018) and computed the amount of actual non-sense reads in each dataset (Table S1-3). Possibility of contamination during library preparation was assessed using blastn and the nt database (Table S4). See supplementary information for further details.

### Computational modules

LongQC consists of several different computational modules. Among them, the coverage module is the core of the pipeline, and it generates coverage statistics and plots that depend on overlap information using a modified version of minimap2 (named minimap2-coverage and see below). See supplementary information for further details.

### Minimap2-coverage and non-sense read definition

To estimate fraction of non-sense reads, overlaps between sampled reads and a given dataset is computed by minimap2-coverage. Output is written in a tab delimited text and is parsed by the coverage module (see above). During overlap calculation, minimap2-coverage filters out some spurious overlaps (supplementary information). Sampled reads having very low coverage (with 2 or fewer overlapping partners as default) are marked as non-sense reads. Overlap calculation also provides extra information in addition to coverage statistics: boundary for adapter like sequences and estimation of per-read error rate. See supplementary information for further details.

### Data availability

LongQC is mainly written in Python3, and it is freely available under the MIT license. The source code and help can be found at https://github.com/yfukasawa/LongQC. The code is compatible with Linux and Mac OS X. Raw sequencing data were deposited in the Sequence Read Archive (SRA) under project PRJNA578920. Supplemental material available at figshare: https://doi.org/10.25387/g3.11516004.

## Results

### Overview of LongQC

LongQC was designed as a quick screening tool to spot issues in TGS data through various statistics and plots: general statistics (yield and noise level), adapter statistics, read length analysis, per-read quality statistics, per-read coverage analysis, GC contents analysis, flanking region analysis, sequence complexity analysis, and per-read sequence error estimation. Combination of these statistics and plots help to understand TGS data and issues, if they exist. Moreover, because all major TGS file formats are supported, users can start QC as soon as data are available. Further details are available in supplementary information with sample plots.

### Quantification of noise in datasets

Sequencing output always comes with a portion of noise such as non-sense reads due to technical or experimental variations in a project. It is important to assure that the noise of sample dataset falls within a certain range. These non-sense reads can be identified and discarded at the stage of read alignment against a reference genome; however, this approach relies on having a high quality reference genome, which might be unavailable. It would be advantageous to estimate the quality of a dataset without a reference genome.

**Figure 1:**
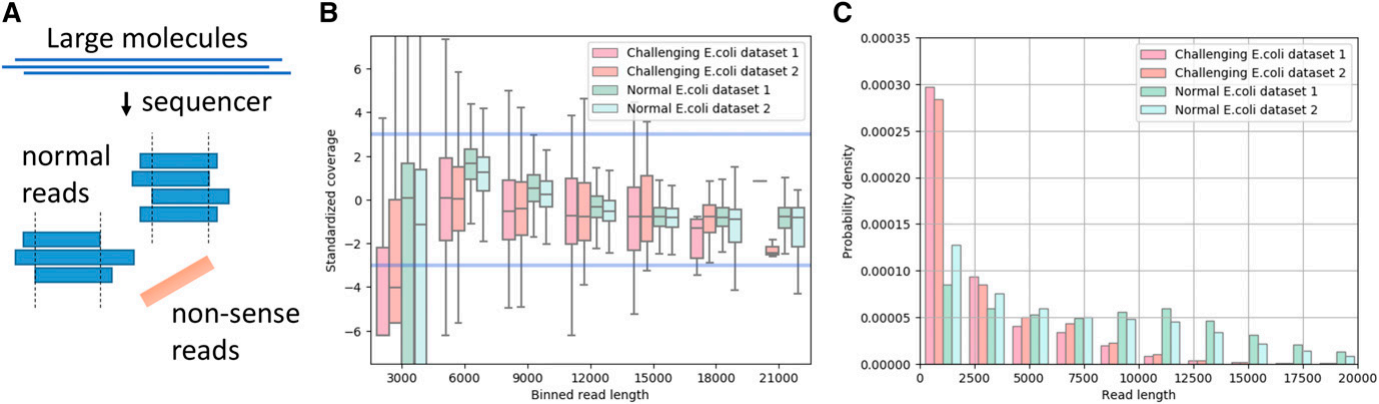
Schematic diagram of non-sense reads and example plots for *E.coli* genome. (A) Blue rectangles represent normal read derived from large molecules such as genomic DNA and orange rectangle shows non-sense read. Non-sense reads have no coverage due to randomness or an even higher error rate. (B) whisker plots for standardized per-read coverage in two challenging and two normal datasets. Standardized per-read coverage is centered by mean of per-read coverage values and divided by standard deviation of per-read coverage values. Blue lines represent 3 standard deviations. (C) read length histograms for the same datasets.

Because the nucleic acids of a sample are fragmented randomly during library preparation, if there is enough coverage, there must be overlaps between reads within a given dataset. With this observation, we designed a QC method without a reference genome using the read overlap concept (Figure 1A).

To put this concept into practical use, quantification of non-sense reads is important. We used publically available and in-house dataset generated on three major platforms to empirically determine non-sense read thresholds (Table S1 and S2). We validated the approach using both simulated datasets and public real datasets. The fraction of non-sense reads estimated by LongQC is very close to the actual fraction in the datasets (Table S3 and S5).

### Assessment using experimentally produced challenging datasets

To assess the capability of LongQC under a real situation, we prepared challenging datasets intended to mimic library failure. Two particularly challenging datasets were generated experimentally on Sequel (supplementary information), which resulted in high non-sense read fractions of 37% and 22%. It is noteworthy that non-sense read fraction is typically lower than 10% in normal PacBio datasets (Table S1). LongQC estimated that 39.6% and 23.4% of these datasets are noise, closely approximating the actual fractions. It shows that; therefore, estimation of the non-sense read fraction gives valuable and timely insight of data quality even in challenging datasets soon after the data acquisition. We noticed that short reads in the challenging datasets show lower coverage; this is observed only in the challenging datasets (Figure 1B). The fraction of very short fragment is higher in the challenging datasets compared to normal datasets (Figure 1C). These suggest that the challenging datasets contain a larger amount of non-sense short fragments. Part of these fragments are short and mappable spiked-in control reads, which would normally be removed during data generation process by Sequel.

We also applied DASCRUBBER, a part of DAZZLER suite. During scrubbing process, for any given dataset, DASCRUBBER computes QVs (quality values) for every short segment and based on the distribution it determines two QV thresholds to define good and bad segments, which would then be used in downstream analysis. A high QV means low quality. Theoretically, threshold values of a low quality dataset are expected to be high compared to those of good quality datasets. Interestingly, while it is possible for DASCRUBBER to notice different proportions of poor-quality reads in our datasets, QV thresholds determined for normal and challenging datasets were comparable and not highlighting any differences (Table S6). Moving forward to downstream analysis using these thresholds might not have paid sufficient attention to the vast quality disparities in these datasets.

### Detection of short fragment contamination

**Figure 2:**
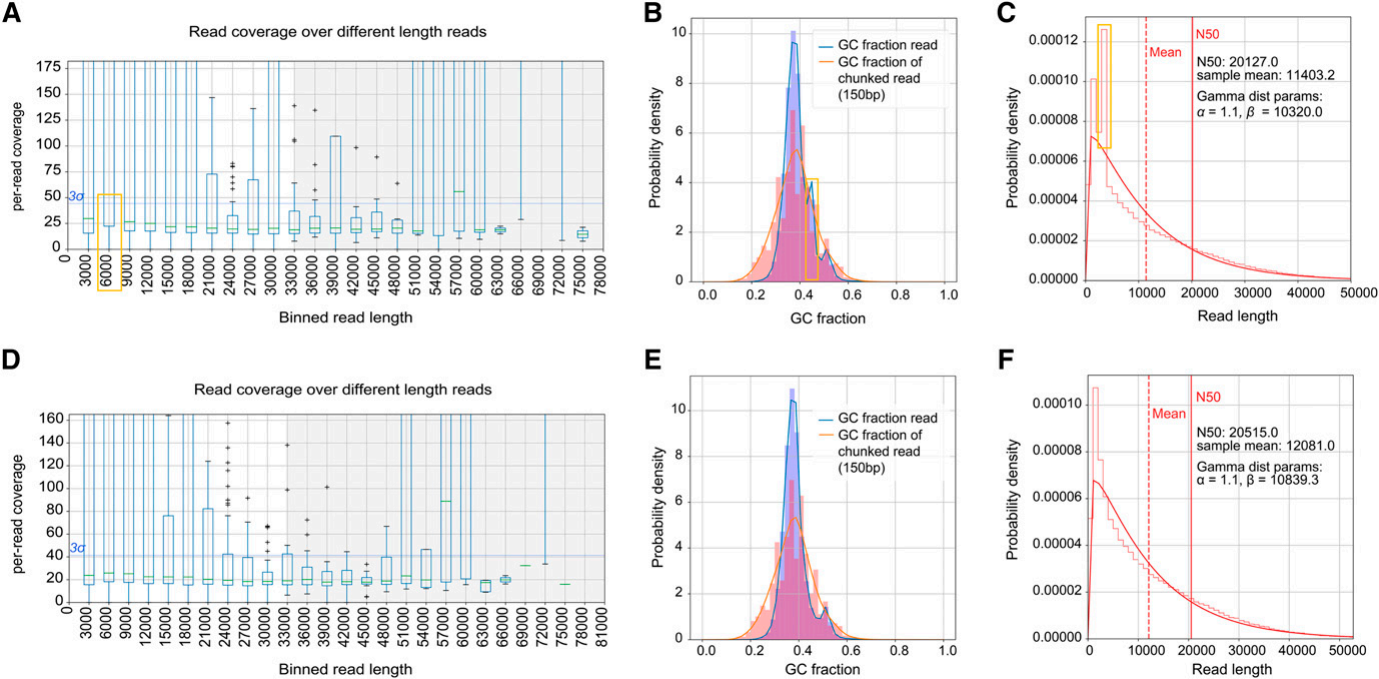
Effects of *E. coli* filter on ONT *A. thaliana* dataset. Top panels were generated from the original dataset and bottom panels show plots after *E. coli* read removal. (A, D) Distribution of per-read coverage. (B, E) GC content distributions. (C, F) Length distributions. Yellow boxes highlighted the spikes that disappeared after *E. coli* read removal.

We tested LongQC on a recently published dataset for *Arabidopsis thaliana* (Michael *et al.* 2018). Interestingly, coverage plot of certain read length (between 3,000 and 6,000 bps) shows fluctuation (Figure 2A), and other plots also have unexpected spikes (Figure 2B,C). Post-hoc analysis revealed that it is highly likely to be resulted from the fact that 8% of this dataset came from a specific region of *E. coli* genome (Table S3). We should note that the spurious spikes in read length, GC content and per-read coverage distribution disappeared by removal of those reads (Figure 2D-F). Although this dataset has an overall high quality, subtle contamination was visualized by LongQC.

### Transcriptomics/Iso-Seq

LongQC is applicable to other types of application such as Iso-Seq on PacBio platform. We applied LongQC on Iso-Seq datasets from humming bird and zebra finch. Without tuning of parameters for overlap computation, non-sense read fractions were estimated accurately (Table S7).

### Empirical assessment of read quality

Assessment of read quality is not trivial when quality values are unavailable. To evaluate read quality in those cases, we slightly modified previously suggested values for read quality assessment using k-mer (Ondov *et al.* 2016; Li 2018). Because reference genomes are not always available, the number of k-mers without any error/mutations is estimated from read to read overlap computations. This modification enabled a rapid computation of empirical read quality without references, and the resulting empirical score was significantly correlated with sequence identity (Table S8). We also validated this formulation using recently reported reads which are likely to be noise but have enough high quality (HQNR), and it was reported that HQNR have no similar sequences in public database (Mojarro *et al.* 2018). This set of reads is expected to be quite random even though they have high quality scores assigned by the sequencer. In fact, HQNR reads show much higher estimated per-read sequence error rate and there is no significant correlation between sequencer based quality and estimated error rate by our formulation (Figure S1. Spearman’s rank correlation = 0.03, p-value = 0.374); therefore, this is consistent with the fact that they cannot be mapped to any known sequences with high confidence (Mojarro *et al.* 2018) (Figure S1). In contrast, there is a strong correlation (Figure S1. Spearman’s rank correlation = -0.90, p-value < 2.2e-16) between our estimated error rates and quality scores assigned by a sequencer for non-HQNR reads (Figure S1). Although there is a small fraction of non-HQNR which have actually high estimated error rates (estimated per-read error rate > 0.2 and per-read QV, an average of quality scores assigned by a sequencer, > 7), we manually confirmed that they have no hits or partial hits against the nt database.

## Discussion

In this study, we have tackled the problem of assessing the quality of dataset generated by TGS, particularly in the absence of a reference genome and/or Phred scores. Existing QC programs are unsuitable or inefficient to process those datasets: the quality of DNA sequences is often evaluated by aligning any query reads against a reference genome, or sequencing files are assessed on quality indicators such as Phred score given by the sequencing platform. However, one or both above conditions can be missing in a TGS dataset, for example, in case of *de novo* assembly without reference or in case of Sequel data where Phred scores are not provided. A platform-independent, computationally efficient and user-friendly QC tool that can operate under these circumstances is needed in order to allow a comprehensive visualization of different characteristics of a dataset and highlight any potential quality issues as a first step right after data acquisition before a deep-dive downstream analysis. It is noticed that there is often a certain amount of reads that cannot be mapped onto any other sequences in the same sequenced library. We call them “non-sense reads” and propose that the fraction of these reads can be used as a powerful indicator of the general data quality.

LongQC is designed to perform quick quality assessment of TGS data and works with all major TGS platforms to date, including PacBio and ONT. Because there is an undeniable trend of sequencing throughputs increasing exponentially, balancing between speed and completeness has become a valid concern even in the QC step. For example, DAZZLER suite is a full analysis package and computational cost could be high, especially for large datasets. While it conducts a thorough all-*vs.*-all comparison, the trade-off is reflected in the computational time, especially for larger normal datasets (Table S10). In contrast, LongQC uses subsampling for a rapid QC before a full analysis without compromising overall usefulness with well-estimated statistics (Table S3 and S5). Users should select appropriate tools to use according to the need and stage of a project.

Long-read technologies are improving rapidly, and may become the mainstay of sequencing in the next decade and it is increasingly popular in various applications to address a diverse range of biological problems. LongQC could be applied on new applications. For example, we tested Iso-Seq datasets for avian transcriptomes and the difference between LongQC-estimated and actual mapping results was small (Table S7). LongQC could also be useful for single cell analysis. Adapter sequences for single cell discrimination can be quite diverse or simply random. Nonetheless, success or failure of such adapter ligations can be interrogated and visualized under LongQC framework before a full analysis (Figure S2).

In conclusion, we showed that LongQC is a valuable, efficient and easy-to-use tool for QC of any present and future TGS data that enables better understanding of the dataset prior to downstream analysis.

## References

[bib1] De Coster W., D’Hert S., Schultz D. T., Cruts M., and Van Broeckhoven C., 2018 NanoPack: visualizing and processing long-read sequencing data. Bioinformatics 34: 2666–2669. 10.1093/bioinformatics/bty149 29547981 PMC6061794

[bib2] Lanfear R., Schalamun M., Kainer D., Wang W., and Schwessinger B., 2019 MinIONQC: fast and simple quality control for MinION sequencing data. Bioinformatics 35: 523–525. 10.1093/bioinformatics/bty654 30052755 PMC6361240

[bib3] Li, H., 2018 Minimap2: pairwise alignment for nucleotide sequences. *Bioinformatics* 10.1093/bioinformatics/bty191 PMC6137996 29750242

[bib4] Loman N. J., and Quinlan A. R., 2014 Poretools: a toolkit for analyzing nanopore sequence data. Bioinformatics 30: 3399–3401. 10.1093/bioinformatics/btu555 25143291 PMC4296151

[bib5] Michael T. P., Jupe F., Bemm F., Motley S. T., Sandoval J. P., 2018 High contiguity Arabidopsis thaliana genome assembly with a single nanopore flow cell. Nat. Commun. 9: 541 10.1038/s41467-018-03016-2 29416032 PMC5803254

[bib6] Mojarro A., Hachey J., Ruvkun G., Zuber M. T., and Carr C. E., 2018 CarrierSeq: a sequence analysis workflow for low-input nanopore sequencing. BMC Bioinformatics 19: 108 10.1186/s12859-018-2124-3 29587645 PMC5872496

[bib7] Ondov B. D., Treangen T. J., Melsted P., Mallonee A. B., Bergman N. H., 2016 Mash: fast genome and metagenome distance estimation using MinHash. Genome Biol. 17: 132 10.1186/s13059-016-0997-x 27323842 PMC4915045

[bib8] Ono Y., Asai K., and Hamada M., 2013 PBSIM: PacBio reads simulator–toward accurate genome assembly. Bioinformatics 29: 119–121. 10.1093/bioinformatics/bts649 23129296

[bib9] Schmieder R., and Edwards R., 2011 Quality control and preprocessing of metagenomic datasets. Bioinformatics 27: 863–864. 10.1093/bioinformatics/btr026 21278185 PMC3051327

[bib10] Watson M., Thomson M., Risse J., Talbot R., Santoyo-Lopez J., 2015 poRe: an R package for the visualization and analysis of nanopore sequencing data. Bioinformatics 31: 114–115. 10.1093/bioinformatics/btu590 25173419 PMC4271141

[bib11] Yang C., Chu J., Warren R. L., and Birol I., 2017 NanoSim: nanopore sequence read simulator based on statistical characterization. Gigascience 6: 1–6. 10.1093/gigascience/gix010 PMC5530317 28327957

