# CORRIGENDUM

**DOI:** 10.1534/g3.120.401778

**Published:** 2020-09-18

In the article by Y. Fukasawa, L. Ermini, H. Wang, K. Carty, and M-S. Cheung (*G3: Genes|Genomes|Genetics* 10: 1193-1196) entitled “LongQC: A Quality Control Tool for Third Generation Sequencing Long Read Data”, on page 1193, Dr. Ming-Sin Cheung’s name was misspelled as “Min-Sin Cheung” and has since been corrected.

